# Clouston Syndrome: Report of a Jordanian Family with GJB6 Gene Mutation

**DOI:** 10.1155/2023/5577379

**Published:** 2023-10-12

**Authors:** Rand Murshidi, Heba Al-lala

**Affiliations:** Department of Dermatology, School of Medicine, University of Jordan, Amman, Jordan

## Abstract

Ectodermal dysplasias (ED) encompass a collection of conditions wherein the development of two or more structures derived from the ectoderm exhibits abnormal patterns. One example of such a rarity within the spectrum of ectodermal dysplasias is hidrotic ectodermal dysplasia, also known as Clouston syndrome. This particular variant is distinguished by a triad of clinical characteristics, which encompass partial-to-complete alopecia, nail dystrophy, and palmoplantar hyperkeratosis. It stands as a scarcely encountered autosomal-dominant inherited disorder, resulting from a mutation in the GJB6 gene that encodes the gap junction protein connexin 30. We hereby document the case of a forty-five-year-old Jordanian woman who presented with alopecia affecting the scalp, eyebrows, and eyelashes, in addition to nail dystrophy. Interestingly, she did not manifest palmoplantar keratoderma. It is worth mentioning that several members of her extended family also manifested similar clinical features. Subsequent genetic testing conclusively established the diagnosis of Clouston syndrome. In light of this diagnosis, comprehensive counseling was extended to the patient.

## 1. Introduction

Ectodermal dysplasia was first documented in 1848, but the term “ectodermal dysplasia (ED)” was not introduced until 1929 [1, 2].

The ectodermal dysplasias are a large group of hereditary disorders characterized by the alteration of ectodermally derived structures. They are categorized into two main groups: hypohidrotic and hidrotic ectodermal dysplasias (Clouston syndrome) [3].

Clouston syndrome is an autosomal-dominant disorder defined by the clinical triad comprising nail dystrophy, generalized hypotrichosis, and palmoplantar hyperkeratosis. The onset of sparse hair and nail dystrophy becomes discernible during the early months of life, potentially culminating in complete alopecia during puberty due to progressive hair loss. In parallel, the development of nail dystrophy follows a gradual trajectory throughout childhood. It is of particular significance to note that clinical presentations can exhibit distinctive variations even among individuals within the same family [4].

This case is being reported to offer the readers a recognition pattern to identify similar rare cases in their own practice.

## 2. Case Presentation

**Figure 1:**
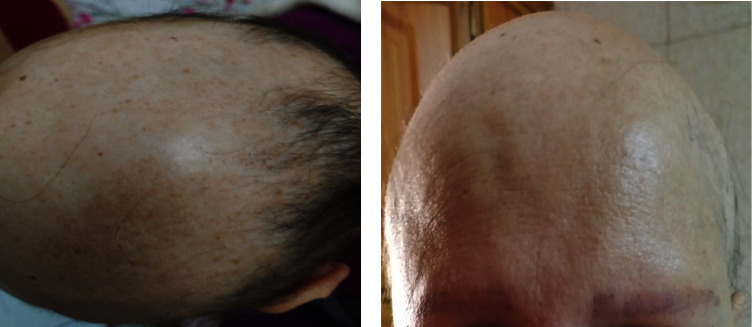
(a) Marked alopecia of the scalp with only sparse hair on the posterior scalp. (b) Alopecia of eyebrows.

**Figure 2:**
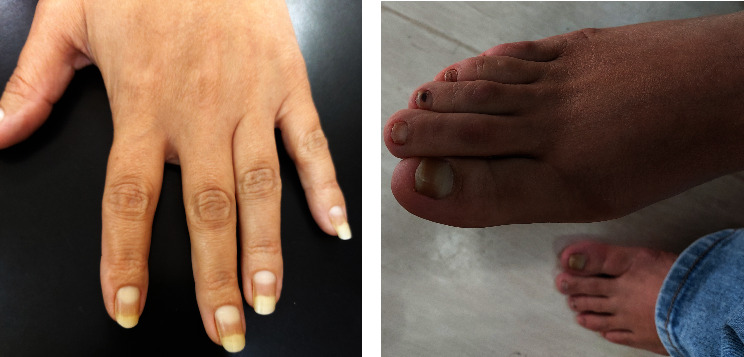
(a) Leukonychia of fingernails. (b) Leukonychia and dystrophy of toenails.

**Figure 3:**
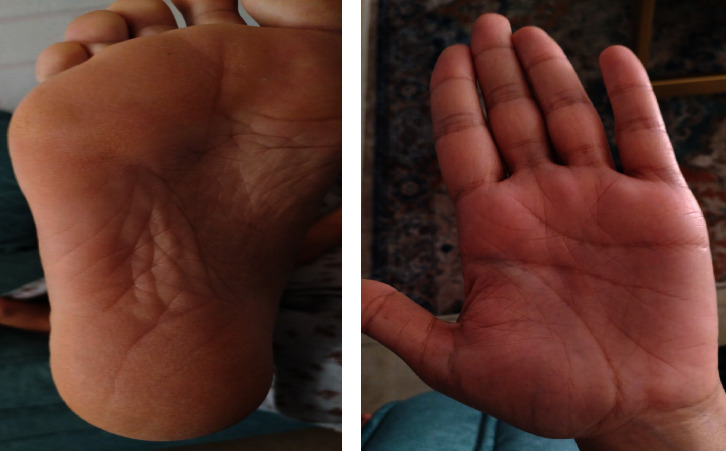
Absence of palmoplantar keratoderma.

A 45-year-old female patient presented to the University of Jordan dermatology clinic with a chief complaint of hair loss affecting the scalp, eyebrows, and eyelashes (Figure 1). She also complained of leukonychia of fingernails, while toenails were more dystrophic (Figure 2). She reported that she used to have sparse hair during her childhood; however, during her twenties, she developed marked alopecia. There was an absence of palmoplantar keratoderma (Figure 3). Upon further questioning, she reported that her father, offspring, and some of her siblings have similar complaints of varying severity.

**Figure 4:**
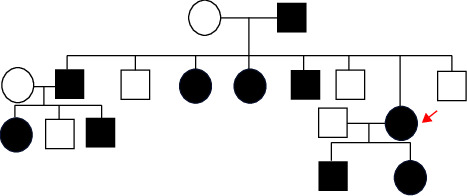
Pedigree of the family. The arrow indicates the proband. The proband's father, progeny, siblings, and the descendants of each were afflicted in an autosomal-dominant pattern, but there was a great variability in the degree of hypotrichosis and nail dystrophy. None of them had palmoplantar hyperkeratosis. Healthy members are indicated by empty symbols. Affected ones are represented by black-filled symbols.

Family history revealed that multiple family members were affected in an autosomal-dominant pattern (Figure 4), but there was a great variability of clinical features.

In the clinic, comprehensive diagnostic procedures were conducted with the aim of assessing potential factors that could contribute to alopecia. These procedures encompassed blood tests designed to evaluate nutritional deficiencies and thyroid function, all of which yielded results falling within the normal range.

Genetic testing of the patient was carried out by the study of the gene panel for ectodermal dysplasias at Igenomix Labs, Spain. A pathogenic variant in heterozygous state was identified in the GJB6 gene; this caused a single nucleotide alteration in the gene's DNA sequence. This alteration led to the substitution of the amino acid glycine with arginine at position 11 in the gap junction protein connexin 30 (Table 1). Notably, p.G11R stands out as the most frequently reported mutation in Clouston syndrome, observed in numerous ethnic populations worldwide [4].

Accordingly, the patient was counseled about her disease mentioning that at present there is no specific treatment and management is purely supportive. Management options include the use of emollients and topical keratolytics (urea or lactic acid based) for softening of the nail plates and palmoplantar hyperkeratosis, the use of physical modalities like wigs for scalp, and tattooing for eyebrows may also be desired [5].

## 3. Discussion

Clouston syndrome represents an autosomal-dominant disorder, marked by the clinical triad of nail dystrophy, generalized hypotrichosis, and palmoplantar hyperkeratosis. The onset of sparse hair and nail dystrophy is evident within the initial months of life, with the potential for complete alopecia during puberty due to progressive hair loss. Concurrently, nail dystrophy gradually develops during childhood. It is noteworthy that clinical manifestations can exhibit variability even among individuals within the same family [4].

Up to this juncture, five distinct mutations within the GJB6 gene have been meticulously documented, all of which display phenotypic traits associated with Clouston syndrome. Among this array of mutations, p.G11R and p.A88V were originally characterized by Lamartine et al. in diverse ethnic populations, while p.V37E was reported by Smith et al. among patients of Scottish descent, and p.D50N was identified by Baris et al. in Israeli patients. Notably, recent findings by Liu et al. unveiled a novel mutation, N14S in GJB6, alongside a mutation, F191L in GJB2, both substantiating their pathogenic role in Clouston syndrome [6].

Of particular significance among these mutations is p.G11R, which has also been identified in the current case report. It stands out for being the most frequently reported mutation and has been observed in diverse ethnic populations worldwide [4]. Furthermore, Clouston syndrome with a recurring heterozygous mutation c.31G > C (p.G11R) within the GJB6 gene was documented by Fujimoto A et al. in a Lebanese-German population in 2003 [7].

It remains uncertain whether a single mutation can account for the genotype-phenotype correlation in all phenotypic variants of hidrotic ectodermal dysplasia. In a Chinese family displaying typical hidrotic ectodermal dysplasia characteristics, the p.G11R mutation was identified [8]. However, it is noteworthy that the same mutation was found in a substantial Indian family diagnosed with Clouston syndrome, marked by the absence of palmoplantar keratoderma [4]. This finding closely aligns with the observations made in the present case report, highlighting the presence of phenotypic heterogeneity.

To conclude, we must think of the possibility of Clouston syndrome that the patient presents with nail dystrophy and generalized hypotrichosis, especially if multiple family members are also affected.

## Figures and Tables

**Table 1:** Genetic test for the patient.

Gene	Variant cHGVS	pHGVS	Type	Zygosity	dbSNP	Interpretation

GJB6	NM_006783.4:c.31G > A	p. Gly11Arg SNV	SNV	Het	NA	Pathogenic

## Data Availability

All data related to the article are available from corresponding authors upon request.

## References

[1] Thurnam J. (1848). Two cases in which the skin, hair and teeth were very imperfectly developed. *Journal of the Royal Society of Medicine*.

[2] Weech A. A. (1929). Hereditary ectodermal dysplasia. Congenital ectodermal defect. *American Journal of Diseases of Children*.

[3] Shah K. N., McKinster C. D. (2020). Ectodermal dysplasia. https://emedicine.medscape.com/article/1110595-overview#showall.

[4] Verma I., Khatter S., Puri R., Mahay S., Bhai P., Saxena R. (2019). Mutation-proved Clouston syndrome in a large Indian family with a variant phenotype. *Indian Journal of Dermatology*.

[5] Lee H. E., Chang I. K., Im M., Seo Y. J., Lee J. H., Lee Y. (2013). Topical minoxidil treatment for congenital alopecia in hypohidrotic ectodermal dysplasia. *Journal of the American Academy of Dermatology*.

[6] Liu Y. T., Guo K., Li J., Liu Y., Zeng W. H., Geng S. M. (2015). Novel mutations in GJB6 and GJB2 in Clouston syndrome. *Clinical and Experimental Dermatology*.

[7] Fujimoto A., Kurban M., Nakamura M. (2013). GJB6, of which mutations underlie Clouston syndrome, is a potential direct target gene of p63. *Journal of Dermatological Science*.

[8] Liao M. Y., Peng H., Li L. N., Yang T., Xiong S. Y., Ye X. Y. (2023). Hidrotic ectodermal dysplasia in a Chinese pedigree: a case report. *World Journal of Clinical Cases*.

